# Innovations and challenges in predicting cognitive trajectories after stroke

**DOI:** 10.1093/braincomms/fcae364

**Published:** 2024-10-16

**Authors:** Nele Demeyere, Margaret J Moore

**Affiliations:** Nuffield Department of Clinical Neurosciences, University of Oxford, Oxford OX3 9DU, UK; Queensland Brain Institute, The University of Queensland, St. Lucia, QLD 4072, Australia

## Abstract

This scientific commentary refers to ‘Deep learning disconnectomes to accelerate and improve long-term predictions for post-stroke symptoms’, by Matsulevits *et al*. (https://doi.org/10.1093/braincomms/fcae338).

**This scientific commentary refers to ‘Deep learning disconnectomes to accelerate and improve long-term predictions for post-stroke symptoms’, by Matsulevits *et al*. (https://doi.org/10.1093/braincomms/fcae338).**

Over recent years, identifying reliable biomarkers that predict stroke recovery outcomes has developed into a key research area within both theoretical and clinically driven research. Developing effective methods to identify the patients most at risk for poor long-term outcomes has the potential to improve clinical care. However, alongside impressive innovations, there are several key challenges that must be met before such prognostication can positively impact clinical care, particularly within the complex topic of post-stroke cognitive impairment.

Artificial intelligence is revolutionizing healthcare research from improved diagnostics to personalized medicine across a multitude of diseases. These advancements are expected to have transformational impacts on patient care. Matsulevits *et al*.,^1^ in their recent article in *Brain Communications*, applied deep-learning models to generate disconnection statistics from brain lesion masks for use in prediction modelling of cognitive outcomes after stroke. This project represents a significant advance as it provides an efficient method for calculating disconnection statistics that are traditionally time-consuming and computationally intensive to generate. Critically, these deep-learning-generated disconnection metrics were found to act as stronger predictors of long-term neuropsychological outcomes compared to traditionally derived disconnection statistics. The authors conclude that this deep-learning approach has the potential to greatly enhance clinical prognostication by allowing clinicians to more easily incorporate disconnection measures into this process.

This high-quality and methodologically rigorous study presents an innovative approach, though current clinical practice is not yet in a place where it can benefit from such advancements. The present clinical reality has several major barriers that are preventing the inclusion of complex indicators (including disconnection statistics) into routine prognostication. These key challenges involve practical limitations and insufficient evidence that complex biomarkers outperform more accessible information sources. However, at its core, there is substantial divergence between how clinical researchers and cognitive neuroscientists understand post-stroke cognitive impairment outcomes. This disconnect results in differing conceptualizations of why the prediction of post-stroke cognitive impairments is needed and how these predictions would ultimately impact clinical decision-making.

First, in terms of practical limitations, clinicians in stroke settings do not generally have access to the lesion masks needed to generate detailed anatomical statistics, including disconnection. While Matsulevits *et al*.^1^ improve the efficiency of disconnectome generation, it is often comparatively more time-consuming to generate the lesion masks that are the required input data. Acute brain imaging is part of standard care in stroke, but generating lesion masks is not. Creating lesion masks generally requires manual delineation and spatial normalization that are time-consuming processes requiring technical expertise and proficiency in specialized software. While recent fundamental research and commercial initiatives are making progress towards developing automated and semi-automated lesion segmentation approaches, manual delineation remains the gold standard. Additionally, many clinical settings only collect MR imaging for a small and select subset of stroke survivors with the majority of patients receiving only comparatively low-quality CT imaging. Future and ongoing innovations into delivering (semi-)automated processes and tools with raw scan inputs can ultimately overcome these practical barriers, which currently preclude the incorporation of complex anatomical statistics in risk prediction modelling.

Second, with regard to comparative predictive value, there are many easily accessible information sources that provide strong and well-established predictors of long-term post-stroke cognitive impairment. A recent large-scale meta-analysis demonstrated that factors such as age and stroke severity are strong predictors of post-stroke cognitive outcome, with additional neuroimaging markers including basic visual ratings of atrophy and white matter disease also identified as key predictors.^2^ However, baseline cognitive assessment performance was by far the strongest predictor of long-term outcomes.^2^ These meta-analytical results in ‘global cognitive’ outcomes are in line with our own findings on more domain-specific cognitive outcomes with the Oxford Cognitive Screen.^3,4^ Ultimately ‘cognition predicting cognition’ is not surprising, and early baseline screening makes for a very cheap and easily accessible addition to predictive modelling. Overall, the majority of clinical research pertaining to predicting cognitive outcomes has focused on predictors that can be easily accessed by clinicians. There is a lack of research directly comparing the prognostic value of complex biomarkers (e.g. disconnection profiles) and established, more easily accessible prognostic indicators. This is an important research gap because for any new sources of predictive information to be incorporated to improve prognostication, they must be demonstrated to provide added information that is not already captured by these more easily accessible variables.

Third, there is a more fundamental issue in the clinical conceptualization of post-stroke cognitive changes. Clinical views are often not in line with how this topic is approached from a cognitive neuroscience angle. Matsulevits *et al*.^1^ quantify cognitive outcomes through a battery of neuropsychological tests and aimed to predict specific test scores from brain data. This approach follows the neuropsychological gold standard using detailed standardized tests to capture potentially subtle deficits. In contrast, clinical research is generally focused on overall diagnostic outcomes, with limited categories. Cognitive outcome is clinically typically considered as the presence versus absence of post-stroke ‘dementia’, or severity of ‘neurocognitive disorder’ as defined by the DSM-5. This type of risk prediction approach aims to flag presence or severity of global cognitive impairment or decline rather than predicting domain-specific impairments that may remain stable or improve over time.^5^ Indeed, the same approaches from Alzheimer’s dementia risk prediction are applied to stroke cohorts, and generally do not consider focal lesion data.^6^

In contrast to this approach, cognitive neuroimaging studies, such as by Matsulevits *et al*.,^1^ aim to predict outcomes in selective, first-time stroke samples without comorbidities. Given that comorbidity rates are very high in stroke (e.g. recurrent stroke, pre-stroke cognitive decline and degenerating brain health), clinically useful prediction models must aim to capture this complexity rather than excluding it from studies. Targeted research is needed to help bridge the gap between these silos of research. The optimal prognostic model will likely need to incorporate a wide range of variables capturing demographics, clinical markers, pre-morbid status, acute cognitive profile, and neuroimaging markers (both brain health and lesion-specific). Given the heterogeneity of stroke, data from large diverse samples will be needed to provide reliable modelling. Multi-centre and multi-disciplinary collaborative endeavours will be necessary to achieve this, however, these comprehensive predictive approaches offer a highly promising avenue for future research.

Finally, it is important to consider the ultimate clinical purpose of individualized prediction outcomes. Optimistically speaking, accurate risk prediction could help refer patients to dedicated neuropsychological rehabilitation that would in turn improve their outcomes. However, the effect of these neuropsychological rehabilitation approaches is generally small and rehabilitation programmes for specific cognitive deficits (e.g. neglect and memory) currently lack the evidence needed to be included in clinical recommendations.^7^ There is a clear need for additional dedicated and high-powered research in rehabilitation. In a context of co-occurring declining brain health, risk prediction modelling of poor outcomes can also help healthcare professionals plan follow-up care, determine discharge destinations and prepare relatives for the onset of dementia. There are no current established clinical practice guidelines outlining how treatment pathways should be different for patients expected to experience poor cognitive outcome. As a minimum, patients expected to have longer term cognitive impairment (either declining or stable) should be flagged for follow-up, with cognition being monitored.^8^

Finally, and importantly, there remain pragmatic questions about how practitioners should frame any clinical decisions and discussions based on individualized prediction outcomes. Though there has been a heavy emphasis within previous research on modelling ‘risk’ and predicting ‘poor outcomes’, informing patients that they are not expected to experience significant recovery and may decline may be anxiety-provoking and may not be helpful or wanted.^9^ Reframing the narrative to emphasize likelihood of recovery, rather than risk of decline or dementia, may support positive psychosocial adjustment for stroke survivors and their family members. The benefits of such complex care pathway interventions will need testing in high-quality (cluster) Randomized trials to establish the evidence required to change clinical practice.

Considered cumulatively, there is a clear need for translational and implementation research to provide guidance on how prognostic indicators can be incorporated to improve stroke care. Meanwhile, fundamental research should continue to establish the building blocks. This includes developing tools that enable clinicians to efficiently access neuroimaging biomarkers, evaluating the effectiveness of neurorehabilitation and conducting large scale research into individual risk and recovery prognostic models combining imaging metrics, acute cognitive performance and demographic and clinical information. To ultimately deliver real-world innovations to improve patient outcomes, we should conceptualize cognitive trajectory prognostics through the lens of a complex intervention framework,^10^ developing and testing individualized cognitive care with an implementation goal from the start, working closely with researchers, practicing clinicians, patients, carers and healthcare governance. This may enable us to bridge the current divide between what is technologically possible, and what is clinically feasible and beneficial with regard to predicting cognitive trajectories after stroke.

## Data Availability

Data sharing not applicable – no new data generated.
